# Relationship between enteral nutrition timing and 28-day mortality in critically ill stroke patients in the MIMIC-IV database

**DOI:** 10.3389/fneur.2024.1411906

**Published:** 2024-08-06

**Authors:** Xiaoliang Wang, Xiuling Xie, Xin Xu, Lan Tan

**Affiliations:** ^1^Neurology Department of Qingdao Municipal Hospital, Nanjing Medical University, Qingdao, Shandong, China; ^2^Neurology Department of Affiliated Hospital of Qingdao University, Qingdao, Shandong, China

**Keywords:** stroke, enteral nutrition, mortality, MIMIC-IV database, intensive care unit (ICU)

## Abstract

**Background:**

The ideal timing for commencing enteral nutrition (EN) in critically ill stroke patients in the intensive care unit (ICU) remains a subject of debate, with ongoing controversy regarding the impact of early EN (EEN) initiation. In this study, we investigated the association between the timing of EN initiation and 28-day mortality using data from the MIMIC-IV database.

**Methods:**

This study employed a retrospective cohort design using the MIMIC-IV database to identify stroke patients who received EN during their hospital stay. The main focus of this investigation was to examine 28-day mortality among these patients following hospital admission. Various demographic, clinical, laboratory, and intervention variables were considered as covariates. The Cox regression analysis was employed to assess the correlation between the timing of EN initiation and 28-day mortality, and restricted cubic splines (RCS) analysis was used to test for non-linear correlation. Patients were then stratified into two cohorts depending on the timing of EN initiation: within 2 days (*n* = 564) and beyond 2 days (*n* = 433). A multivariate Cox regression analysis was used to investigate the difference in 28-day mortality between the groups.

**Results:**

A total of 997 participants were included in this study, with 318 (31.9%) dying within 28 days. We observed that the timing of EN initiation correlated with 28-day mortality, but this correlation was not significant after adjusting for covariates (crude HR: 0.94, 95% CI: 0.88–1, *p* = 0.044; adjusted HR: 0.96, 95% CI: 0.9–1.02, *p* = 0.178). The RCS analysis showed that the correlation was not non-linear. Notably, in the multivariate regression models, early EN initiation was associated with a higher mortality rate compared to late EN initiation [odds ratio (OR) = 1.34, 95% CI: 1.06–1.67, *p* = 0.012]. After adjusting for various confounding factors in the multivariate Cox regression models, we identified that patients in the early EN group had a 28% higher risk of mortality than those in the reference group (OR = 1.27, 95% CI: 1–1.61, *p* = 0.048). These associations remained consistent across various patient characteristics, as revealed through stratified analyses.

**Conclusions:**

Early commencement of EN in critically ill stroke patients may be linked to a higher risk of 28-day mortality, highlighting the need for further investigation and a more nuanced consideration of the optimal timing for commencing EN in this patient population.

## Background

Stroke is a major global health concern and is responsible for causing a substantial number of fatalities and disabilities (1). In recent years, China alone has reported millions of new stroke cases and related deaths (2). Critically ill stroke patients often experience reduced consciousness levels, severe dysphagia, and impaired gastrointestinal function, making them susceptible to malnutrition (3, 4). Malnourishment or nutritional vulnerability in stroke patients is associated with higher mortality rates, increased complications, and a poor functional prognosis (3–5). Guidelines suggest that critically ill stroke patients with reduced consciousness levels or prolonged severe dysphagia receive early enteral tube feeding within 72 h from the onset of symptoms (6, 7).

However, the optimal timing for initiating EN in severe stroke cases is still debated. Some studies have shown improved outcomes with early tube feeding within 7 days (8), while others suggest that initiating enteral nutrition within 3 days in comatose stroke patients does not improve nutritional status and may increase the likelihood of diarrhea (9). Consequently, this study aims to provide further insights into the relationship between the timing of enteral nutrition initiation and 28-day mortality in severe stroke patients, utilizing data from the MIMIC-IV database.

## Methods

### Study population

The present retrospective study utilized patient data from the Medical Information Mart for Intensive Care-IV (MIMIC-IV) cohort, a single-center, longitudinal cohort spanning from 2008 to 2019. The MIMIC-IV database contains various patient information, including demographics, vital signs, laboratory results, diagnoses, medication details, and follow-up information. The data extraction code is available on GitHub (10) (http://github.com/MIT-LCP/mimic-iv). This study adhered to the Strengthening the Reporting of Observational Studies in Epidemiology (STROBE) guidelines (11).

### Inclusion and exclusion criteria

This study included stroke patients, both ischemic and hemorrhagic, who received enteral nutrition (EN) during their initial ICU admission. Stroke diagnoses were based on ICD-9 or ICD-10 (International Classification of Disease, Ninth and Tenth Versions), with stroke consistently listed as the primary diagnosis. The inclusion criteria were as follows: (1) patients aged 18 years or older; (2) those with an ICU stay lasting 24 h or more; and (3) only patients in their initial ICU stay were considered. The exclusion criteria were as follows: (1) patients younger than 18 years, (2) patients with an ICU stay of fewer than 24 h, (3) patients with contradictions to enteral nutrition, such as gastrointestinal bleeding or intestinal obstruction, and (4) patients with a wait time for enteral nutrition beyond 15 days.

### Data extraction

Structured Query Language (SQL) was used for data extraction. The following variables, collected within 24 h of ICU admission, were extracted:

1. **Patient's basic information**: Sex, admission age, race, admission time, ICD code, EN start time, height, weight, body mass index (BMI), and 28-day mortality.
2. **Vital signs**: Temperature, mean blood pressure (MAP), heart rate, and SpO2.
3. **Severity of illness scores**: The Sequential Organ Failure Assessment (SOFA) score, the Simplified Acute Physiology Score II (SAPS II), the Glasgow Coma Scale (GCS), and the Charlson comorbidity index. If a patient was intubated and could not provide a verbal score for the GCS, it was estimated from the eye and motor scores, as reported in previous studies (12).
4. **Laboratory test results**: WBC count, creatinine levels, hematocrit (HCT), and albumin levels.
5. **Treatment modalities**: Mechanical thrombectomy and thrombolysis.
6. **Commodities**: Myocardial infarction, peripheral vascular disease, dementia, COPD, malignant cancer, renal disease, cancer, severe liver disease, gastrointestinal bleeding, or intestinal obstruction, identified by ICD codes.

### Variable definition and outcomes

EN initiation time was defined as the duration between the start of enteral nutrition (EN) and the time of ICU admission. We calculated the initiation time for various EN solutions, including Ensure, Impact, and Vivonex, among others. The main objective of this study was to investigate 28-day mortality as the primary outcome.

### Statistical analysis

Baseline patient characteristics were stratified based on different EN initiation time groups. Continuous data are expressed as either the mean ± standard deviation or median (inter-quartile), while categorical variables are expressed as numbers (percentages), as appropriate. Statistical comparisons between the two groups included the use of the chi-square test or Fisher's exact test for categorical variables, and continuous variables were examined using the analysis of variance test or rank-sum test.

We omitted the missing data for glucose levels, GCS, WBC, body weight, and creatinine due to the low percentage of missing data (missing rate below 5%). We also used multiple imputations to handle the missing data for BMI (missing rate: 34.9%) and serum albumin levels (missing rate: 47.8%).

We selected confounders based on clinical relevance and their association with the outcome (*p* < 0.2) or a change in effect estimate of more than 10%. Multivariable Cox regression models were introduced to evaluate the association between early EN initiation and 28-day mortality. Four models were employed in the regression analysis, each adjusted for different sets of variables:

Model 1: Adjusted for sex and admission age.

Model 2: Adjusted for stroke type, BMI, and GCS in addition to variables in Model 1.

Model 3: Adjusted for mechanical ventilation, the Charlson comorbidity index, serum albumin level, glucose, SAPS II, and MAP in addition to variables in Model 2.

Model 4: Adjusted for malignant cancer, diabetes, myocardial infarction, creatinine, WBC, platelets, HCT, thrombolysis therapy, and mechanical thrombectomy in addition to variables in Model 3.

To explore potential non-linear relationships between the timing of EN initiation and 28-day mortality, a restricted cubic spline (RCS) was utilized. Heterogeneity across subgroups was assessed using Cox regression analysis, and interactions were evaluated using a likelihood ratio test.

Statistical significance was determined using a two-tailed test with a threshold of *P* of < 0.05. All data analyses were conducted using the R software package (R version 4.0.3) and Free Statistics software version 1.7.

## Results

### Baseline characteristics

**Figure 1:**
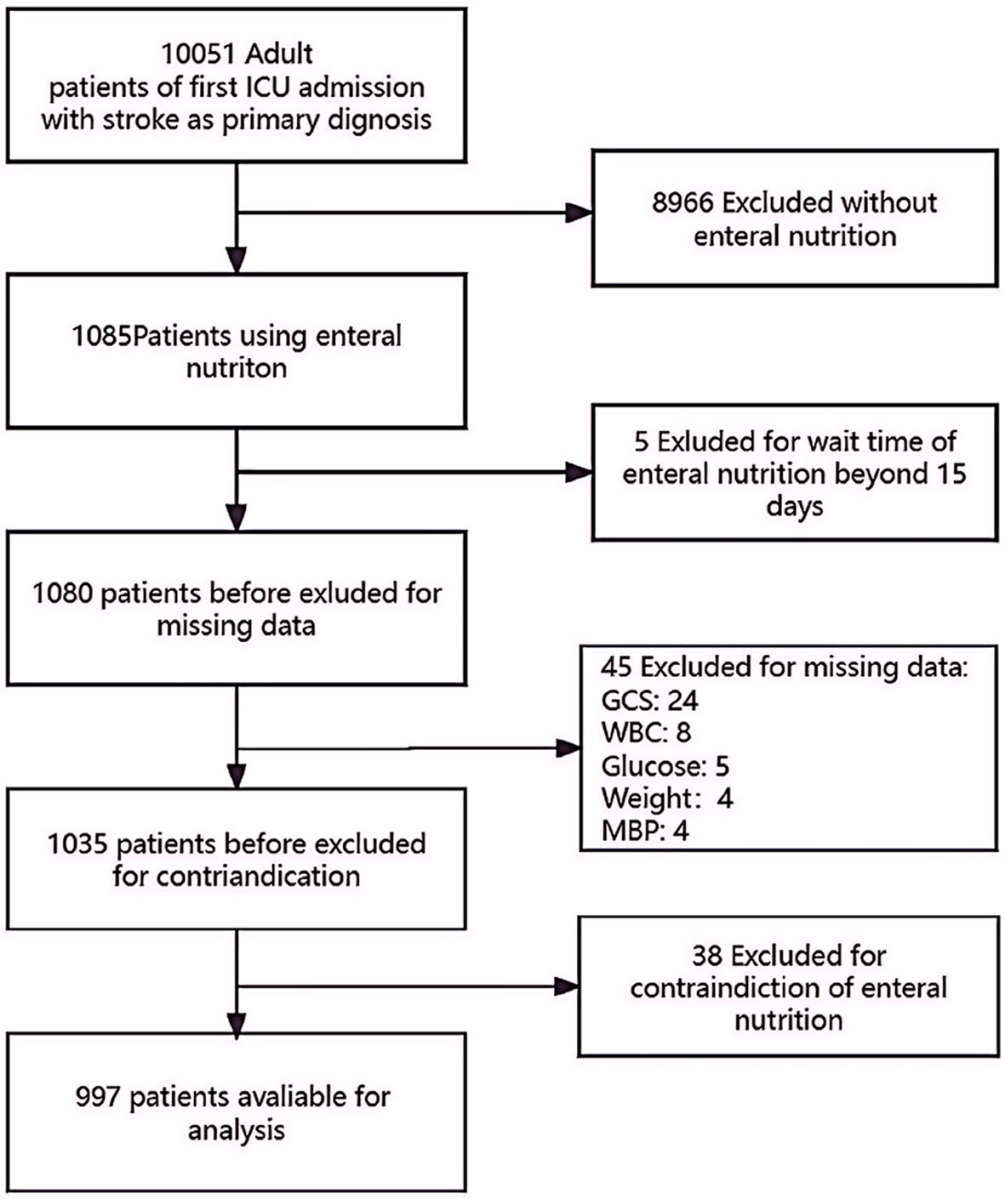
A detailed flowchart illustrating the participant recruitment process. GCS, Glasgow Coma Scale; WBC, white blood cell count; ICU, intensive care unit.

A total of 10,051 individuals with a primary diagnosis of stroke, either ischemic or hemorrhagic, were identified and admitted to the ICU. Among them, 1,085 patients received enteral nutritional (EN) support. After excluding cases with outlier data (where the wait time for EN exceeded 15 days), contraindications, and missing data, our final cohort consisted of 997 stroke patients who received EN. Of these patients, 318 (31.9%) died within 28 days of their hospital admission. Figure 1 provides a detailed flowchart illustrating the participant recruitment process.

The average age of the 997 patients was 69.4 ± 14.6 years, and nearly half of them were women (49.9%). Table 1 presents the baseline characteristics of the included individuals. Compared to the late EN cohort, the early EN cohort had older patients, a higher prevalence of hemorrhagic stroke, a lower prevalence of mechanical ventilation, and lower admission albumin levels.

**Table 1:** Clinical information, and process parameters in patients categorized by EN start-time.

	**Total (*n* = 997)**	**Late EN group (*n* = 433)**	**Early EN group (*n* = 564)**	***p***
**Demographics**				
Sex *n* (%)				0.826
Female	498 (49.9)	215 (49.7)	284 (50.4)	
Male	499 (50.1)	218 (50.3)	280 (49.6)	
Age, mean (SD), y	69.4(14.6)	67.4(14.9)	71.0(14.2)	**< 0.001**
BMI, kg/m^2^	27.2(6.4)	27.1 (6.2)	27.3(6.5)	0.671
**Vital signs, mean (SD)**				
Heart_rate, bpm	81.1 (13.5)	81.5 (13.9)	80.8 (13.1)	0.391
MAP, mmHg	86.0(10.3)	86.0(10.6)	85.9(10.0)	0.881
**Lab test, mean (SD)**				
HCT	38.5 (5.5)	38.4 (5.5)	38.6 (5.6)	0.621
Platelets, 10^9^/L	236.2 (92.3)	240.8 (98.9)	232.7 (86.8)	0.166
WBC, 10^9^/L	13.1 (5.3)	13.2 (5.6)	13.1 (5.0)	0.629
Albumin, g/dL	3.8 (0.6)	3.9 (0.6)	3.7 (0.6)	**0.002**
Glucose, mg/dL	140.2 (40.4)	139.4 (39.1)	140.9 (41.3)	0.569
Creatinine, mg/dL	1.2 (1.1)	1.3 (1.3)	1.2 (1.0)	0.397
**Commodities**, ***n*** **(%)**				
Myocardial infarct	106 (10.6)	52 (12)	54 (9.6)	0.216
Diabetes	234 (23.5)	103 (23.8)	131 (23.2)	0.878
Malignant cancer	56 (5.6)	24 (5.5)	32 (5.7)	0.929
**Severity score, mean (SD)**				
Charlson comorbidity index	6.8 (2.5)	6.6 (2.6)	6.9 (2.5)	0.128
SAPSii	35.9 (10.8)	35.5 (11.4)	36.2 (10.3)	0.37
GCS	8.1 (3.1)	8.0 (3.1)	8.2 (3.0)	0.178
**Stroke sub-type**, ***n*** **(%)**				**0.015**
Ischemic stroke	370 (37.1)	179 (41.3)	191 (33.9)	
Hemorrhagic stroke	627 (62.9)	254 (58.7)	373 (66.1)	
**Interventions**, ***n*** **(%)**				
Thrombolysis	92 (9.2)	37 (8.5)	55 (9.8)	0.514
Mechanical thrombectomy	96 (9.6)	37 (8.5)	59 (10.5)	0.309
Mechanical ventilation	682 (68.4)	313 (72.3)	369 (65.4)	**0.021**
EN start-time	2.4 (2.0)	3.9 (2.1)	1.2 (0.5)	**< 0.001**
**28 days Mortality**, ***n*** **(%)**	318 (31.9)	121 (27.9)	197 (34.9)	**0.019**

SAPS, simplified acute physiology score; MAP, mean arterial pressure; HCT, Hematocrit; WBC, white blood cell count;. GCS, Glasgow coma scale; EN, enteral nutrition; SD, standard deviation.

Bold values indicate statistical significance (*p* < 0.05).

### Effects of EN initiation time on 28-day mortality

In our univariate Cox regression analysis (see Table 2), we observed a decline in 28-day mortality with increasing EN initiation time. Specifically, for each additional day of delay in EN initiation, there was a 6% decrease in the odds of death within 28 days [odds ratio (OR) = 0.94, 95% CI: 0.88–1, *p* = 0.044]. However, this correlation was not significant after adjusting for covariates.

**Table 2:** Univariate logistic regression evaluating the association between baseline characteristic and 28 days-mortality.

**Variable**	**OR_95CI**	***P***
Early EN	1.34 (1.06–1.67)	**0.012**
EN start_time	0.94 (0.88–1)	**0.044**
Female	0.94 (0.75–1.17)	0.582
Admission_age	1.03 (1.02–1.04)	**< 0.001**
BMI	0.9933 (0.976–1.0109)	0.453
GCS	0.84 (0.81–0.87)	**< 0.001**
Hemorrhagic stroke	0.86 (0.69–1.08)	0.198
Thromblysis	0.97 (0.66–1.43)	0.873
Mechanical thrombectomy	0.82 (0.55–1.22)	0.332
Mechanical ventilation	1.75 (1.34–2.27)	**< 0.001**
MAP	0.99 (0.98–1)	0.054
Glucose	1.0066 (1.0042–1.009)	**< 0.001**
Hematocrit	0.99 (0.97–1.01)	0.299
Hemoglobin	1 (0.94–1.08)	0.902
Platelets	0.9999 (0.9987–1.0011)	0.894
WBC	1.04 (1.02–1.06)	**< 0.001**
Albumin	0.66 (0.55–0.79)	**< 0.001**
Creatinine	1.17 (1.09–1.25)	**< 0.001**
Myocardial_infarct	1.46 (1.06–2)	**0.019**
Diabetes	1.12 (0.87–1.44)	0.383
Malignant cancer	1.69 (1.14–2.51)	**0.009**
SAPSII	1.04 (1.03–1.05)	**< 0.001**
Charlson comorbidity index	1.15 (1.11–1.2)	**< 0.001**

SAPS, simplified acute physiology score; MAP, mean arterial pressure; HCT, Hematocrit; WBC, white blood cell count; GCS, Glasgow coma scale. Bold values indicate *p* < 0.05.

On the other hand, the early EN group had a 34% higher mortality rate than the reference group (OR = 1.34, 95% CI: 1.06–1.67, *p* = 0.012). After adjusting for various confounding factors in the multivariable Cox models (see Table 3), we found that patients in the early EN group had a 27% higher risk of mortality than those in the reference group (OR = 1.27, 95% CI: 1.−1.61, *p* = 0.048).

**Table 3:** Multivariable COX regression models evaluating the association between early EN and 28-days mortality.

**Model**	**crude.OR_95CI**	**crude.*P*_value**	**adj.OR_95CI**	**adj.*P*_value**
Model 1	1.34 (1.06–1.67)	0.012	1.23 (0.98–1.54)	0.074
Model 2			1.32 (1.05–1.66)	0.018
Model 3			1.29 (1.02–1.63)	0.032
Model 4			1.27 (1–1.61)	0.048

Model 1: Adjusted for sex and age.

Model 2: Adjusted for sex, age, stroke type, BMI and GCS.

Model 3: Adjusted sex, age, stroke type, BMI, GCS, Mechanical ventilation, Charlson comorbidity index, serum albumin level, glucose, SAPSii, MAP.

Model 4: Adjusted sex, age, stroke type, BMI, GCS, mechanical ventilation, Charlson comorbidity index, serum albumin level, glucose, SAPSii, MAP, malignant cancer, diabetes, myocardial infarction, creatinine, WBC, platelets, HCT, thromblysis therapy, mechanical thrombectomy.

**Figure 2:**
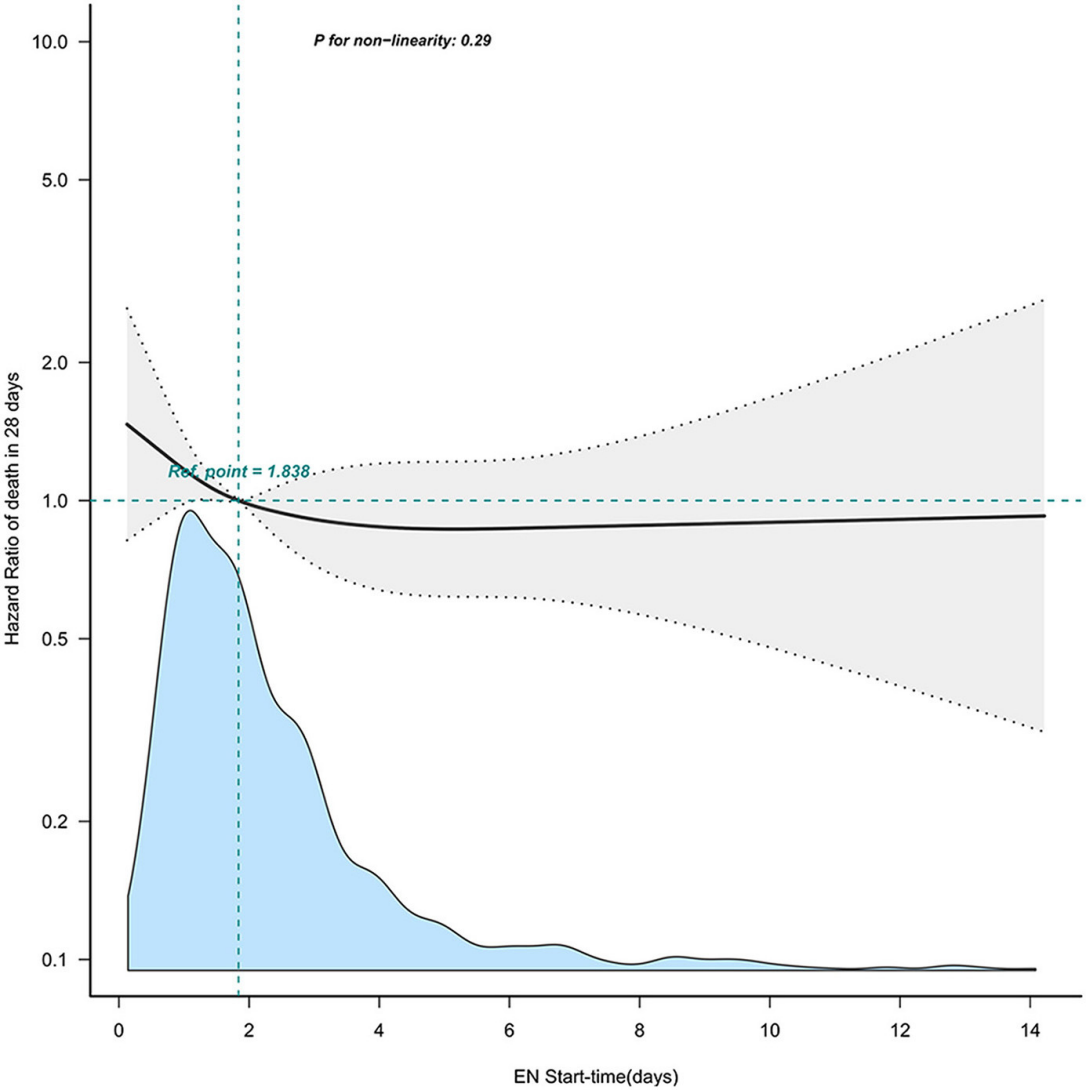
The non-linear relationship between EN start time and 28-day mortality. Adjusted confounders in model 4. This curve illustrates the mortality hazard of different EN start times within our cohort, using the median value of 1.8 days as a reference point. EN, enteral nutrition.

We performed multivariable-adjusted restricted cubic spline analyses to further explore the association between EN initiation time and 28 days mortality. These analyses did not indicate a non-linear relationship between EN initiation time and mortality (as depicted in Figure 2).

**Figure 3:**
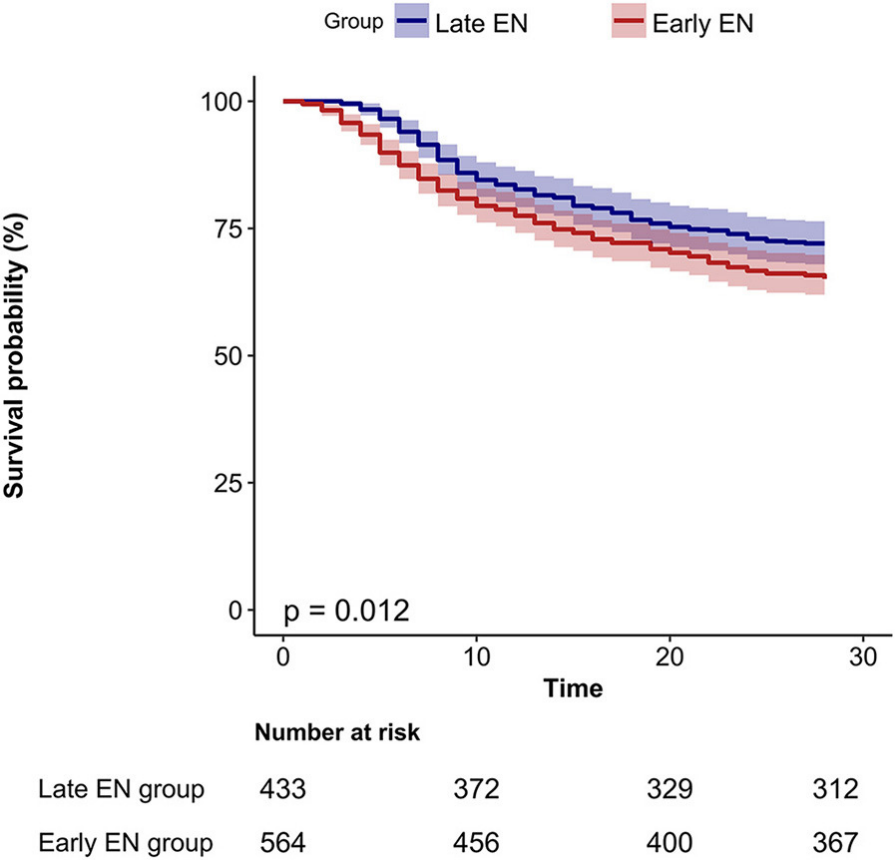
The K-M curve of survival in different EN (enteral nutrition) start time groups. EN, enteral nutrition.

Survival analysis of these two groups showed that patients in the early EN group had poorer overall survival compared to those in the late EN group (*p* = 0.012) (Figure 3).

### Sensitivity analysis

**Figure 4:**
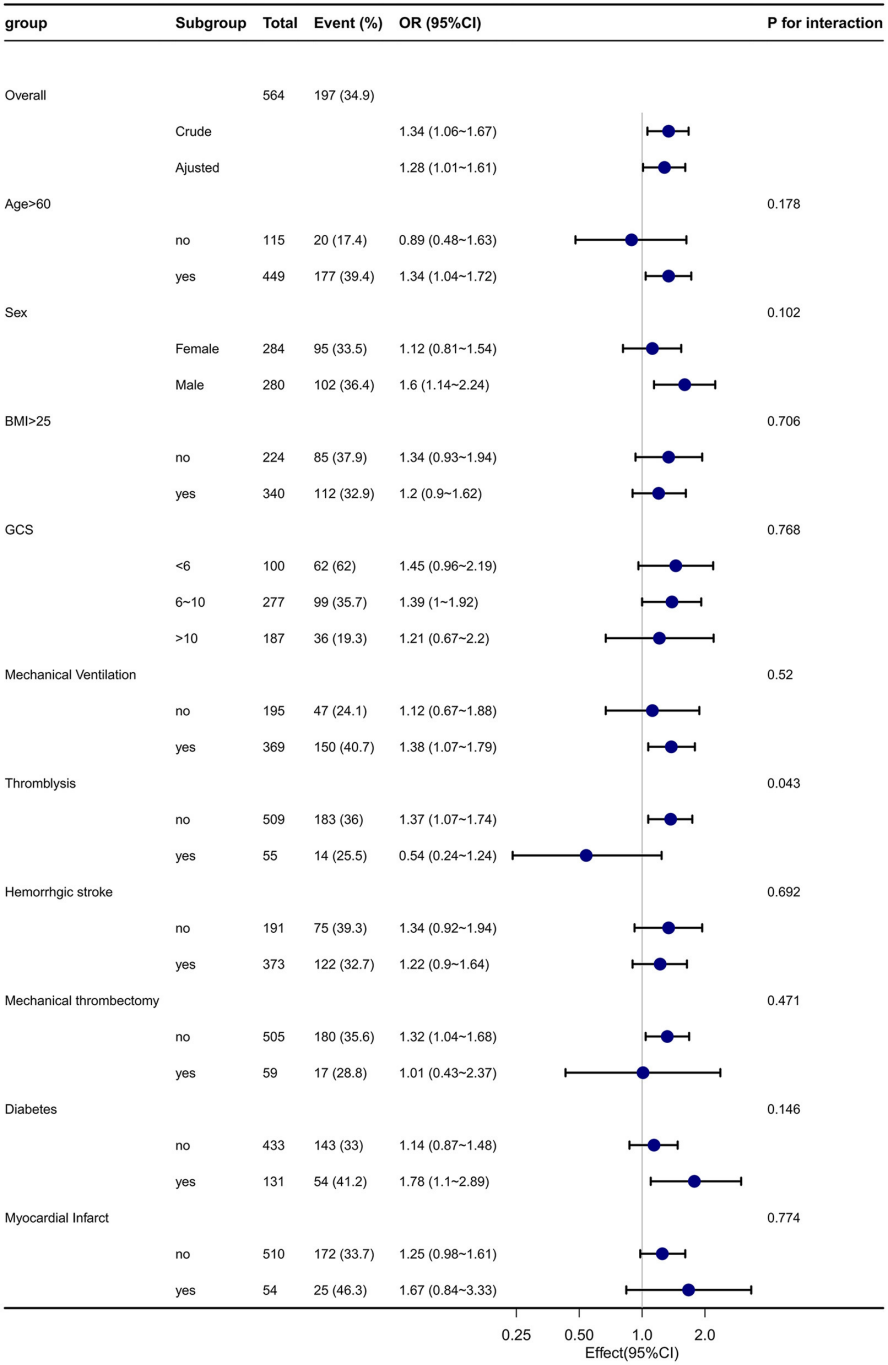
Effect size of early EN on 28-day mortality in each subgroup. A subgroup with varied demographics, including age, sex, Glasgow Coma Scale (GCS) scores, BMI, comorbidities such as diabetes and myocardial infarction, and different interventions, was analyzed within the early EN (enteral nutrition) group (*n* = 564), and the late EN (enteral nutrition) group (*n* = 433) was shown as a reference. The results from this subgroup analysis have demonstrated consistent and robust findings. GCS, Glasgow Coma Scale; WBC, white blood cell count; BMI, body mass index; SAPSii, Simplified Acute Physiology Score II; MAP: mean blood pressure Adjusted Charlson comorbidity index, SAPS II, MAP, creatinine, albumin, WBC, platelets, hematocrit, and glucose.

In our multivariable Cox models, we meticulously adjusted for various confounding factors to assess the relationship between early EN initiation and 28-day mortality. Notably, the association remained consistent across all models (see Table 3). To further investigate the robustness of our findings, we conducted subgroup analyses based on various confounding factors such as age, sex, GCS, BMI, stroke type, thrombolysis, and thrombectomy (see Figure 4). We found a significant interaction in the subgroup of patients who underwent thrombolysis, which may be attributed to the small sample size in this group. No significant interactions were detected within any of the other subgroups (all *p* > 0.05) (Figure 4).

To mitigate potential bias, we redefined the early EN group as involving patients who started EN within the first day of admission (*n* = 211) and the late EN group as those who started EN after 1 day of admission (*n* = 768). After meticulous adjustments for various confounding factors in our multivariable Cox models, participants in the early EN group exhibited a higher risk of mortality (OR = 1.24, 95% CI: 0.96–1.61, *p* = 0.117) (Table 4).

**Table 4:** Multivariable COX regression model evaluating the association between early EN (in 1 day) and 28 days mortality.

**Variable**	***n*.total**	***n*.event_%**	**crude.OR_95CI**	**crude.*P*_value**	**adj.OR_95CI**	**adj.*P*_value**
EN after 1 day	768	239 (30.4)	1 (Ref)		1 (Ref)	
EN in 1 day	211	79 (37.4)	1.35 (1.05–1.74)	0.02	1.24 (0.95–1.61)	0.117

Adjusted confunders in Model 4.

We then divided the patients into four groups, EN within 1 day (*n* = 211), at 1–2 days (*n* = 353), 2–3 days (*n* = 209), and beyond 3 days (*n* = 224), to explore potential dose-response relationships. We found that that groups receiving EN in 2–3 days and beyond 3 days were associated with lower morality (all *p* < 0.05) compared to the early EN group (those receiving EN in 1 day), while the 1–2 days group did not show a significant difference in morality (*p* = 0.26) (Table 5).

**Table 5:** Multivariable COX regression model evaluating the association between different EN initiation days and 28 days mortality.

**EN initiation days**	***n***	***n*.event_%**	**Follow-up.Time**	**crude.HR_95CI**	**crude.*P*_value**	**adj.HR_95CI**	**adj.*P*_value**
≤ 1 day	211	79 (37.4)	4,445	1 (Ref)		1 (Ref)	
1–2 days	353	118 (33.4)	7,969	0.83 (0.63–1.11)	0.206	0.85 (0.63–1.13)	0.263
2–3 days	209	58 (27.8)	4,808	0.68 (0.48–0.95)	0.026	0.71 (0.5–1)	0.05
>3 days	224	63 (28.1)	5,347	0.66 (0.47–0.92)	0.013	0.7 (0.5–0.99)	0.044
Trend.test	997	318 (31.9)	22,569	0.86 (0.78–0.96)	0.006	0.88 (0.79–0.99)	0.026

Adjusted confunders in Model 4.

## Discussion

This study primarily focused on investigating the correlation between the timing of enteral nutrition (EN) initiation and short-term outcomes in stroke patients admitted to the ICU. Our analysis revealed an independent correlation between EN initiation time and 28-day mortality, suggesting that starting EN within 2 days may be associated with higher mortality, which differs from mainstream views in this field of research.

These findings emphasize the crucial role of EN initiation timing in determining short-term outcomes for stroke patients receiving enteral nutrition in the ICU. It is essential to carefully consider the optimal time to initiate EN to minimize adverse outcomes. Early enteral nutrition (EN) offers advantages over total parenteral nutrition (TPN) in treating critically ill patients (13). It helps maintain gastrointestinal integrity, prevents intestinal bacterial translocation (14–19), and enhances recovery during the early hyper-metabolic stage (17, 19). It is evident from recent research that early EN is linked to positive outcomes, including lower mortality and fewer infectious complications, in patients with traumatic brain injury and intracranial hemorrhage (13, 18, 19).

However, it is crucial to emphasize that initiating intragastic feeding too early may result in elevated gastric load, hinder gastric emptying, and increase the chances of aspiration pneumonia in critically ill neurological patients (20). This finding has sparked significant debates regarding the ideal timing for commencing EN (21).

A recent meta-analysis comparing early parenteral nutrition (EPN) to early enteral nutrition (EEN) showed that EPN was more effective in lowering mortality and decreasing infectious complications in patients with acute gut-intolerant phase traumatic brain injuries (21). However, in the case of stroke patients, randomized controlled trials did not show any impact on mortality when comparing early and delayed EN (22).

Furthermore, a study conducted by Yamada et al. found that initiating enteral nutrition too early in comatose stroke patients may not provide nutritional advantages compared to early administration of total parenteral nutrition (TPN), and it might increase the risk of diarrhea (9).

Despite the limited number of studies addressing early EN and its impact on mortality, Cai et al. discovered that administering early EN within 48 h significantly reduced the occurrence of chronic hydrocephalus in severe intracranial hemorrhage patients (23). Similarly, Choi et al. reported that EEN within 72 h was associated with lower 28-day mortality and a reduced occurrence of infectious complications in critically ill neurological patients (24). It is worth noting that the definition of EEN varied among these studies, and our research specifically focused on stroke patients in ICUs.

The potential mechanisms underlying the increased 28-day mortality associated with EEN in critically ill stroke patients remain unclear. However, several possible explanations can be speculated upon. First, initiating early intro-gastric feeding has been associated with an increased likelihood of experiencing gastric complications and developing aspiration pneumonia in critically ill patients with brain injuries (20). These complications can negatively affect patient outcomes and contribute to the observed increase in mortality.

Second, the sympathetic hyperactivation resulting from increased intracranial pressure caused by a stroke can negatively affect gastrointestinal function (25–27). This disruption in the autonomic nervous system may interfere with proper gastrointestinal functioning, leading to complications and potentially higher mortality rates in stroke patients receiving EEN.

Additionally, when comparing EPN and EEN in patients with traumatic brain injuries, research has indicated that EPN is more effective in reducing mortality and infectious complications during the acute gut-intolerant phase (21). This finding suggests that the choice of feeding method can significantly impact patient outcomes, and in certain critical conditions, EEN may not be as effective.

### Strengths and limitations

Our study boasts several strengths. First, it investigated the association between early enteral nutrition (EN) start time and 28-day mortality in stroke patients admitted to the ICU, providing novel insights into the field. We used RCS to test for non-linear correlations between EN start time and outcomes, which contributed valuable information for the development of EN therapy management strategies for this patient population.

Second, we conducted multiple sensitivity analyses to ensure robustness. Subgroup analyses were conducted based on stroke subtypes, age, sex, GCS score, and thrombolytic/thrombectomy therapy. The consistent results across these subgroups indicate that the relationship between EN initiation time and 28-day mortality remains independent of these factors. An interaction was found in the subgroup of thrombolysis, which may be due to the small sample size, this interaction requires further investigation. Additionally, our Cox regression analyses, adjusted across multiple models, accounted for confounding factors and validated the robustness of the outcomes.

However, our study has limitations. First, the unavailability of the National Institutes of Health Stroke Scale (NIHSS) score in the MIMIC-IV database prevented its inclusion as an important predictor of stroke severity (9). Nonetheless, the GCS score served as an alternative measure of neurological function.

Second, during the wait time for EN, only five patients receive TPN in our cohort, and we did not account for TPN in our analysis. Additionally, the calories received before EN could not be acquired from the MIMIC-IV database.

Third, detailed information such as whether EN feeding was successful was difficult to obtain from the MIMIC-IV database, even though the success rate of feeding is an important factor in 28-day mortality. This limitation could introduce bias into our findings.

Fourth, our study was limited to the United States and a single ICU institution, potentially impacting the generalizability of the findings. Different healthcare settings and practices might influence the applicability of the findings. Future prospective multicenter studies could help validate these results in a more diverse patient population.

Finally, considering that a particular stroke population (9.9%) was analyzed, there may be a selection bias that could affect the results. Conducting future randomized controlled trials would provide more robust evidence to validate these findings.

## Conclusion

In conclusion, this study revealed an increased risk of 28-day mortality when EN was initiated within the first 2 days for stroke patients admitted to the ICU. Further research is imperative to understand the significance of this association. Subsequent research confirming these findings could establish a theoretical basis for delineating a specific time window to implement targeted nutritional therapy strategies for stroke patients.

## Data availability statement

The data utilized in this study were obtained from the Medical Information Mart for Intensive Care IV (MIMIC-IV) Clinical Database (28). Requests for access to these datasets could be directed to PhysioNet (29, 30) and the raw data supporting the conclusions of this article will be made available by the authors, without undue reservation.

## Ethics statement

Ethical review and approval was not required for the study on human participants in accordance with the local legislation and institutional requirements. Written informed consent from the patients/participants or patients/participants' legal guardian/next of kin was not required to participate in this study in accordance with the national legislation and the institutional requirements.

## Author contributions

XW: Writing – original draft, Software, Investigation, Formal Analysis, Data curation, Conceptualization. XXi: Writing – original draft, Visualization, Supervision, Resources, Investigation, Formal Analysis, Data curation. XXu: Writing – review & editing, Visualization, Validation, Supervision. LT: Writing – review & editing, Validation, Supervision, Methodology, Conceptualization.
